# Angiomyolipoma of the Nasal Cavity

**DOI:** 10.1590/S1808-86942011000200021

**Published:** 2015-10-19

**Authors:** Mariana Dória Moreira, Marcus Miranda Lessa, Clara Mônica Fiqueredo de Lima, Hélio Andrade Lessa, Luciano Espinheira Fonseca Júnior

**Affiliations:** 1 Resident Physician - Prof. Edgard Santos University Hospital (HUPES)-UFBa; 2 PhD in Sciences - ENT Department - University of São Paulo, Associate Researcher - ENT and Immunology Services - HUPES-UFBa; 3 MSc student in Health Sciences - UFBa, Endoscopic Nasal Surgery Fellow - HUPES-UFBa; 4 PhD in Surgery - Medical School - Federal University of Bahia, Head of the ENT Department -HUPES-UFBa; 5 PhD in Human Pathology - UFBA - FIOCRUZ, Adjunct Professor - Pathology Service - HUPES-UFBa. Hospital Universitário Prof. Edgard Santos - Universidade Federal da Bahia

**Keywords:** angiomyolipoma, nasal cavity, video-assisted surgery

## INTRODUCTION

The angiomyolipoma (AML) is a hamartoma tumor, most of the times of kidney origin, having the liver as the second most common site; often times associated with tuberous sclerosis. Angiomyolipomas originating from other sites are much rarer, and there are very few descriptions of cases in the mediastinum, heart, spermatic cord, vaginal wall, Fallopian tube, oral cavity, pharynx, nasal cavities and the skin^1^, ^2^. Nonetheless, there are important differences between these and the angiomyolipoma arising from the kidneys or liver which will be discussed later.

We found only seven reports of this tumor arising from the nasal cavities in the literature; ours would be the eighth case.

## CASE PRESENTATION

One male, 54 year-old patient came to our ENT service in 2006, complaining of 20 years of spontaneous and recurrent epistaxis, always on the left side. These were quick and self-contained episodes, which happened from one to two times per month and had worsened in the last year. He did not have nasal obstruction, hyposmia, rhinorrhea or local injury.

**Figure 1:**
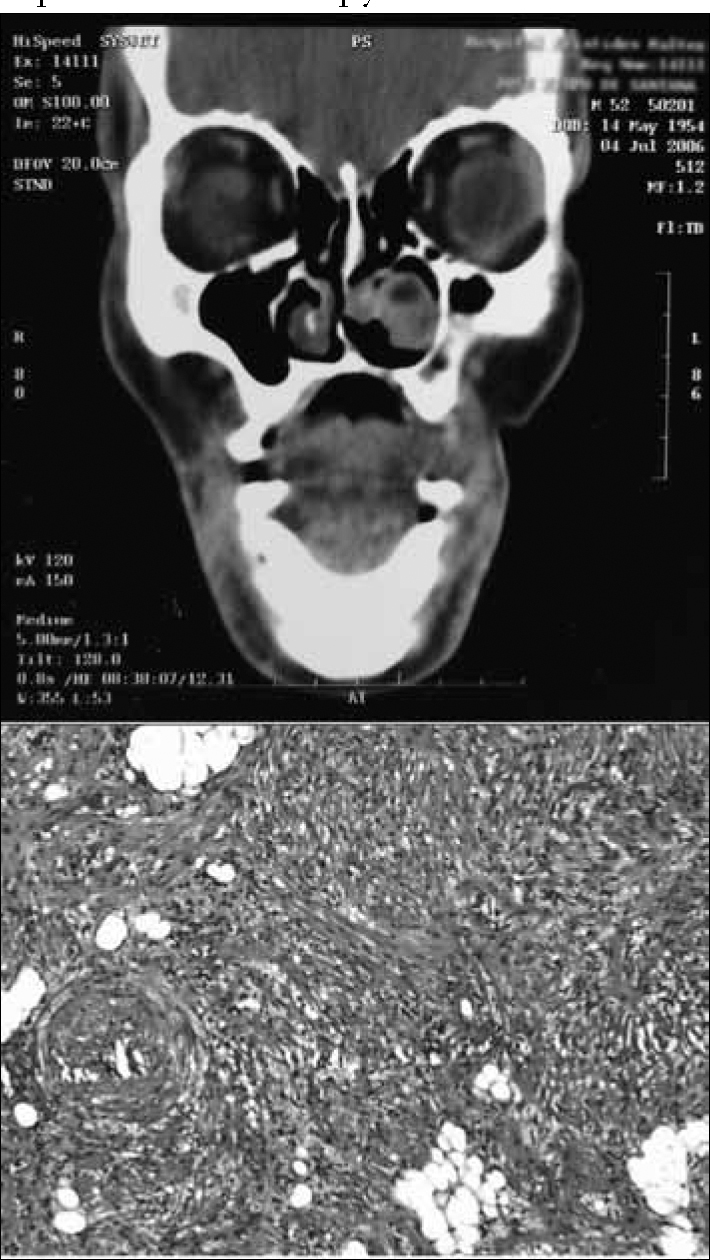
ABOVE: CT scan showing a tumor in the left inferior meatus, without signs of bone destruction. BELOW: Smooth muscle fibers, fat tissue without atypias and thick-wall vessels. H&E 40X.

Upon nasal endoscopy we noticed a round, red and smooth surface lesion in the anterior region of the left inferior meatus. The right nasal cavity and the remaining of the physical exam were normal. We ordered a CT scan of the paranasal sinuses which showed a soft tissue lesion in the left inferior meatus, shifting medially to the inferior turbinate, towards the nasal septum, without signs of bone erosion (Fig. 1). The patient did not have comorbidities, including tuberous sclerosis or diabetes mellitus (DM).

He was submitted to nasal endoscopic surgery in October of the same year, in which we found a macroscopically round, smooth lesion in the left inferior meatus, near the outlet of the nasolacrimal duct, and the lesion was resected without complications.

Pathology revealed a tumor made up of well-differentiated typical leiomyocites, pervaded by proliferated vessels with thick and twisted walls, besides well differentiated fat tissue, suggesting angiomyolipoma (Fig. 1). HMB-45 immunohistochemistry was negative.

The patient remains in periodic follow up in our clinic, He has been asymptomatic and he does not have changes upon nasal endoscopy.

## DISCUSSION

The kidney angiomyolipoma (AML) is a hamartoma tumor made up of smooth muscle, vessels and fat tissue in different ratios. It is closely associated with the occurrence of tuberous sclerosis (TS), and about 80% of patients with TS have AML and half of the kidney AML patients have TS^3^, ^4^. AML may occasionally affect the liver, which is the second most common site of this disease, and it is apparently not associated with tuberous sclerosis^3^, ^4^, ^5^.

Of all the AML described from sites other than the kidney and liver, the most commonly found are those on the skin. Skin AML has been described on the penis, head and neck and limbs. Histopathologically speaking, the skin and nasal AML are identical, as are the oral cavity and pharyngeal types. Nonetheless, these have important pathology differences from kidney and liver AML; and Watanabe & Suzuki^3^ proposed a joint denomination of mucocutaneous AML for this second group of tumors.

Mucocutaneous AML are different in many aspects from those arising from the kidneys or liver. First, they are not associated with TS, they usually affect older men and are usually small, contrary to what happens to kidney and liver tumors, which are, often times, large. However, the most striking difference is that the nasal cavity and skin AML are made up only of mature smooth muscle cells which are negative to the HMB-45 melanoma-specific antigen, contrary to those which are not mucocutaneous.

Of the 7 patients with nasal cavity AML found in the literature, 5 were men and 2 were women. Mean age was 45 to 88 years and three in seven (42%) had DM associated. Our patient follows such incidence, an adult young man without diabetes.

## FINAL REMARKS

AML is a very rare tumor, especially among those which affect the nasal cavity. Nonetheless, it is necessary to be attentive to the possible differential diagnosis in nasal cavity tumors, especially when facing a unilateral nasal tumors.
